# Possible impact of neutrophils on immune responses during early pregnancy in ruminants

**DOI:** 10.1590/1984-3143-AR2021-0048

**Published:** 2021-10-27

**Authors:** Mariani Farias Fiorenza, Carolina dos Santos Amaral, Adriana Raquel de Almeida da Anunciação, Valério Valdetar Marques Portela, Mohammed Ali Marey, Akio Miyamoto, Alfredo Quites Antoniazzi

**Affiliations:** 1 Programa de Pós-graduação em Medicina Veterinária, Universidade Federal de Santa Maria, Santa Maria, RS, Brasil; 2 Global Agromedicine Research Center, Obihiro University of Agricultural and Veterinary Medicine, Obihiro, Japan; 3 Department of Theriogenology, Faculty of Veterinary Medicine, Damanhour University, Damanhour, Egypt

**Keywords:** innate cells, interferon tau, inflammation, cattle

## Abstract

The interaction between early embryo and maternal immune system for the establishment of pregnancy is the focus of several studies; however, it remains unclear. The maternal immune response needs to keep a balance between avoiding any damage to the conceptus and maintaining its function in combating microbes as well. When conceptus-maternal crosstalk cannot achieve this balance, pregnancy losses might occur. Intercommunication between mother and conceptus is fundamental during early pregnancy to dictate the outcome of pregnancy. In ruminants, the embryo reacts with the maternal system mainly *via* interferon tau (IFNT) release. IFNT can act locally on the embryo and endometrial cells and systemically in several tissues and cells to regulate their response *via* the expression of interferon-stimulated genes (ISGs). Also, IFNT can induce the expression of inflammatory-related genes in immune cells. Day 7 embryo induces a shift in the maternal immune response towards anti-inflammatory (Th2) immune responses. During maternal recognition of pregnancy, peripheral mononuclear cells (PBMCs) and polymorphonuclear cells (PMNs) express markers that configure an anti-inflammatory response. However, PMNs response is more sensitive to the effects of IFNT. PMNs are more likely to express interferon-stimulated genes (ISGs), transforming growth factor-beta (TGFB), interleukin 10 (IL10), and arginase-1 (ARG1), configuring one of the most rapid immune responses to early pregnancy. This review focus on the local and peripheral immune responses during early pregnancy in ruminants, mainly the PMNs function in the immune system.

## Introduction

Pregnancy represents one of the most critical periods for species conservation; therefore, it is essential to understand the mechanisms that protect the dam and its offspring (Leber et al., 2010). The maternal recognition of pregnancy (MRP) period culminates with the maximum gestational losses in cows. The period with more impact on reproduction may vary according to the individual; however, high-production dairy cows concentrate their losses around Day 8 after fertilization (Diskin et al., 2011; Sartori et al., 2002; Wiebold, 1988). These embryonic losses derive from several factors and generate a profound economic impact (Diskin et al., 2011). The solution for this problem may have a substantial effect on the reproductive performance of the global herd.

In ruminants, the central MRP signaling molecule is type I interferon (IFN) tau (IFNT), which acts in a paracrine manner in the uterus, together with progesterone (P4), stimulating the production of histotroph by the endometrial glands, providing nutrition to the embryo, consequently its development (Brooks et al., 2014; Spencer et al., 2007, 2004). IFNT immunological role can be linked with immune cell recruitment (Imakawa et al., 2005), lymphocyte proliferation inhibition (Skopets et al., 1992), natural killer (NK) cells stimulation (Tuo et al., 1993). Also, IFNT can modulate gene expression in endometrial epithelial cells and immune cells. Many of these genes are interferon-stimulated genes (ISGs) and immune response genes (Walker et al., 2010), possibly to generate an immune-tolerant environment for the embryo’s development.

Polymorphonuclear cells (PMNs) are the first line of defense of the organism against an aggressor agent. They were the immune system’s main villains for a long time due to their characteristics (Elliott et al., 2017). However, the first cells to migrate to the injury site showed remarkable plasticity to establish highly specialized processes, such as pregnancy (Fridlender et al., 2009). IFNT modulates PMN responses by inducing ISGs and immune response-related genes (Walker et al., 2010) to modulate the maternal immune response. Pregnancy-related factors can modulate PMN phenotype to maintain embryonic and fetal development (Kropf et al., 2007; Ssemaganda et al., 2014). Immune cells exchange factors to modulate the response according to the situation, e.g., PMNs can modify lymphocyte phenotype to a more tolerogenic type to help pregnancy development (Nadkarni et al., 2016). Therefore, a successful pregnancy depends on the maternal immune system’s ability to change and adapt to each specific developmental stage. Therefore, this review aimed to discuss the immune response, mainly neutrophils, during maternal recognition of ruminants’ pregnancy.

### Interferon tau (IFNT)

Pregnancy goes through a critical phase (Degrelle et al., 2005) named MRP (Short, 1969). The embryo secretes factors acting autocrine, paracrine, and endocrine (Godkin et al., 1984; Oliveira and Hansen, 2008; Wang et al., 2013). Although the first stages of uterine remodeling and implantation seem to be programmed by maternal hormones regardless of the presence of the embryo (Sandra et al., 2015), pregnancy requires conceptus-maternal crosstalk before implantation to generate an MRP signal and regulate gene expression of different cell types (Forde and Lonergan, 2017). Initially, the embryo communicates to the mother as early as Day 4 (Talukder et al., 2018). It is possible to detect IFNT effects around Day 7 in the uterus (Sponchiado et al., 2017). Additionally, there is a biochemical modulation of the uterine environment by the embryo on Day 7, possibly to help prepare the endometrium for pregnancy (Sponchiado et al., 2019). This communication by embryonic signals vary according to mammal species, and IFNT is considered the MRP signal in ruminants (Imakawa et al., 1987; Short, 1969).

Maternal recognition of pregnancy is the mechanism by which the embryo signals to the maternal system to help maintain pregnancy (Geisert et al., 1988; Roberts et al., 2008; Short, 1969; Spencer et al., 2007; Vallet et al., 1988). Early pregnancy recognition befalls through the secretion of IFNT (Imakawa et al., 1987) by trophoblastic cells (Farin et al., 1989). The concentration of IFNT can be detected around Day 15 in the systemic circulation (Han et al., 2006), and its production ceases at the beginning of implantation (Demmers et al., 2001). Usually, the MRP occurs early to maintain the corpus luteum (CL) for production of P4 throughout pregnancy in cows (Bazer et al., 1986; Thatcher et al., 1986) by inhibiting the luteolysis process (Martal et al., 1998; Roberts et al., 2008).

Type I IFNs belong to a family of cytokines that have a critical role in linking innate and adaptive responses to protect and immunomodulate the organism against viral infection (González-Navajas et al., 2012). IFNT has vital antiviral, antiproliferative, and immunomodulatory activities (Roberts, 1989). Besides, IFNT stimulates the expression of ISGs probably to protect the uterine environment and embryo against viral infections (Bazer and Thatcher, 2017) and help in the development of tolerance of the maternal response to the semi allogenic concept, i.e., half of its genetic material is from paternal inheritance (Billingham et al., 1953). IFNT gene has a homology of 70% with IFNO, 50% with IFNA, and 25% with IFNB in cattle (Leaman and Roberts, 1992). IFNT is distinguished from other IFNs by its trophoblast-specific, time-specific, and constitutive transcriptional control variables (Ezashi and Imakawa, 2017). Nearly every cell type, including leukocytes, fibroblasts, and endothelial cells, can produce another type I IFNs. Depending on the stimulus and the responding cell types, the signaling pathways that lead to the induction of type I IFNs vary but lead to the activation of some common signaling molecules (Häcker et al., 2006). Diverse compounds, like double-stranded RNA, induce IFNA and IFNB for just a few hours (Khabar and Young, 2007; Whittemore and Maniatis, 1990). IFNT, on the other hand, is not influenced by viruses or double-stranded RNA and is produced for more than several days (Farin et al., 1991). Besides that, IFNT shows antiproliferative and antiviral activities with less toxicity than IFNA (Pontzer et al., 1988; Subramaniam et al., 1995).

Furthermore, the metabolism, transport, and density of prostaglandins (PGs) and their receptors also appear to be influenced by IFNT (Arosh et al., 2004), suggesting that inadequate endometrial response to IFNT may be one of the reasons for gestational failure (Asselin et al., 1997). IFNT acts in an autocrine manner in the trophoblast cells to help embryo development (Brooks and Spencer, 2015; Wang et al., 2013), paracrine in the endometrium luminal epithelium to avoid luteolytic pulses of prostaglandin F2 alpha (PGF) and prepare the endometrium for pregnancy (Spencer and Bazer, 1996), and endocrine action manner in extrauterine cells to signalize the pregnancy to the mother (Oliveira et al., 2008).

Interferon tau is one of the main factors in the conceptus-maternal crosstalk. Its action results in the rescue of CL, immune cell activation, and recruitment (Bai et al., 2012; Bazer et al., 2015; Hansen et al., 2017). Studies have found significant antiviral activity in the uterine vein’s blood 15 and 16 days after conception (Bott et al., 2010; Oliveira et al., 2008; Romero et al., 2015). IFNT enters the uterine vein and, as a result, stimulates the expression of multiple ISGs and immune response genes in blood cells to help maintain pregnancy (Green et al., 2010; Oliveira et al., 2008; Shirasuna et al., 2012; Talukder et al., 2019). Collectively, in addition to the modulatory effects on embryo development, endometrial and luteal environments, IFNT also coordinates the maternal immune response during the MRP (Hansen et al., 2017).

### Immunological changes during maternal recognition of pregnancy

Successful mammalian pregnancy is partly dependent on the release and action of various cytokines and other immunomodulators by conceptus-maternal unit (Billingham et al., 1953) and crosstalk between innate and adaptive immune cells (Arck and Hecher, 2013). During gestational development, immunological patterns change regulated by conceptus signaling to boost communication with the maternal system (Mor and Cardenas, 2010). When the whole pregnancy is analyzed, these patterns can alternate between a pro- or anti-inflammatory state. These changes rely on specific mediators (Mor and Abrahams, 2002; Romero et al., 2006), like IFNT during the MRP (Ott et al., 2014).

Early pregnancy establishment requires an anti-inflammatory or Th2 type environment, and a sudden shift to a pro-inflammatory Th1 type immune response could lead to complications in pregnancy (Reinhard et al., 1998; Wegmann et al., 1993). However, current research argues against this notion and has shown a Th1 type environment in healthy pregnancies (Germain et al., 2007; Gupta et al., 2005). The Th1 type immune response is characterized by the secretion of IFNG, interleukin (IL) 1 beta (IL1B), IL2, IL15, IL18, and tumor necrosis factor-alpha (TNFA). In contrast, Th2 type immune response has a more significant concentration of IL4, IL5, IL6, IL10, IL13, and granulocyte-macrophage colony-stimulating factor (GM-CSF) (Mosmann et al., 1986; Raghupathy, 1997; Wilczyński, 2005).

Extensive changes occur in gene expression of pregnant animals’ cells, especially ISGs and immune response-related genes (Bauersachs et al., 2006; Walker et al., 2010). Pregnant ewes had a higher expression of ISGs in the thymus (Zhang et al., 2020), higher expression of IL5, and a lower expression of IFNG, IL2, IL4, IL6, and IL10 in the liver (Yang et al., 2019). The expression of anti-inflammatory factors, such as IL10 and transforming growth factor-beta (TGFB) and pro-inflammatory like TNFA, were reported in endometrial and immune cells in cows (Rashid et al., 2018; Shirasuna et al., 2012; Talukder et al., 2017).

The secretion of IL10 by a diverse set of maternal and conceptus cells helps to orchestrate normal pregnancy. IL10 is a significant player in directing cell differentiation towards a Th2 phenotype (Thaxton and Sharma, 2010), inhibiting the production of PGs and cytokines and regulating macrophage activation (Svensson et al., 2011). TGFB may play a significant role in controlling apoptosis and cell survival at specific stages of pregnancy (Shooner et al., 2005), inhibiting proliferation and differentiation of lymphocytes and the activation of other leukocytes (Letterio and Roberts, 1998), and inducing differentiation of neutrophils towards an anti-inflammatory phenotype (Mishalian et al., 2013). IL4 can induce differentiation of naive T cells into Th2 cells and suppress the production of Th1 cells and IFNG (Le Gros et al., 1990; Tanaka et al., 1993). IL5 is known to be beneficial for normal pregnancy (Makhseed et al., 1999). IL6 is a pro- and anti-inflammatory cytokine associated with pregnancy tolerance, helping embryo-maternal crosstalk and implantation (Blitek et al., 2012; Prins et al., 2012). TNFA can be related to inflammatory mechanisms related to implantation, placentation, and pregnancy outcome (Alijotas-Reig et al., 2017). Also, TNFA levels were higher in the CL of pregnant than non-pregnant cows, perhaps to help CL formation and maintenance (Sakumoto et al., 2014). IFNG is a classical Th1 cytokine that can suppress the Th2 response by enhancing the shift of naive T cells into Th1 cells (Nakagome et al., 2009). Another Th1 cytokine is IL2 that may lead to infertility (Bilotas et al., 2015).

Endometrial stromal macrophages and dendritic cells start expanding around Day 13 of pregnancy and may characterize the maternal immune response to the developing embryo in cattle (Mansouri-Attia et al., 2012). IFNT administration reduced the peripheral circulation of T helper cells, B cells, and gamma delta T cells without changing the number of T cytotoxic cells (Tuo et al., 1999). The number and recruitment of T regulatory cells to the endometrium also increased; complementary abnormal pregnancy is associated with T regulatory cell function inhibition. T regulatory cells secrete IL4 and induce tolerance to paternal alloantigen, helping develop a pregnancy (Aluvihare et al., 2004). Also, IFNT and IFNA dose-dependently decreased lymphocyte proliferation (Fair, 2016; Skopets et al., 1992) and changed the number, distribution, and activity of NK cells on Day 16 of pregnancy (Oliveira et al., 2013). An essential component from the innate immune response that is mainly changed on Day 18 of pregnant heifers is the pattern recognition receptors (PRR) (Rocha et al., 2021), which suggest that these proteins are not only modulated during sperm recognition by the female immune system (Akthar et al., 2020; Elesh et al., 2021; Ezz et al., 2019) but also has an essential role during early pregnancy (Rocha et al., 2021). This review will focus on studies that provided the importance of PMNs during pregnancy.

### Maternal recognition of pregnancy and neutrophils

Neutrophils are the main population of immune cells that provide the first defense line during infection to ensure returning to the physiologic state (Mayadas et al., 2014). These cells migrate to the damaged area and phagocyte, degranulate and destroy the foreign body (Basu et al., 2000; Pillay et al., 2013). The classical denomination of neutrophils is short-lived cells with three main primary activities: 1) the production and release of granules, 2) oxidative molecules (Heifets, 1982), and 3) neutrophil extracellular traps (NETs) (Takei et al., 1996). Once neutrophils migrate into damaged tissues, a complex bidirectional interaction with immune and non-immune cells starts (Mantovani et al., 2011). Neutrophils can modulate the immune system, regulate hematopoiesis, angiogenesis, and wound healing, in addition to their functions (Kolaczkowska and Kubes, 2013; Mantovani et al., 2011; Pruijt et al., 2002; Tecchio and Cassatella, 2014).

Depending on the hormonal profile, the endometrium of a cow undergoes numerous changes during the estrous cycle. PMN infiltration increases into the endometrium, especially from proestrus to metestrus (Ohtani et al., 1993). During these phases, PMNs represent the most common phagocytic cells in the uterus (Skjerven, 1956). In pregnancy, PMNs can detect implantation (Kizaki et al., 2013; Manjari et al., 2016; Shirasuna et al., 2012), causing neutrophils to be recruited and activated in a specific way allowing proper placentation and angiogenesis to occur (Hannan and Salamonsen, 2007). Furthermore, PMNs showed a slight decrease in number and activity in successful pregnancy implantation. Following that, their number and activity were maintained throughout the pregnancy (Mohammed et al., 2017). Problems like delayed apoptosis in normal pregnancy can promote persistent inflammation and contribute to pregnancy-associated neutrophilia and pregnancy-induced inflammatory changes in the peripheral blood neutrophils (Gilbert, 2011). An increase in neutrophil number and inflammatory activity may result in infertility or pregnancy loss (Mohammed et al., 2017).

**Figure 1:**
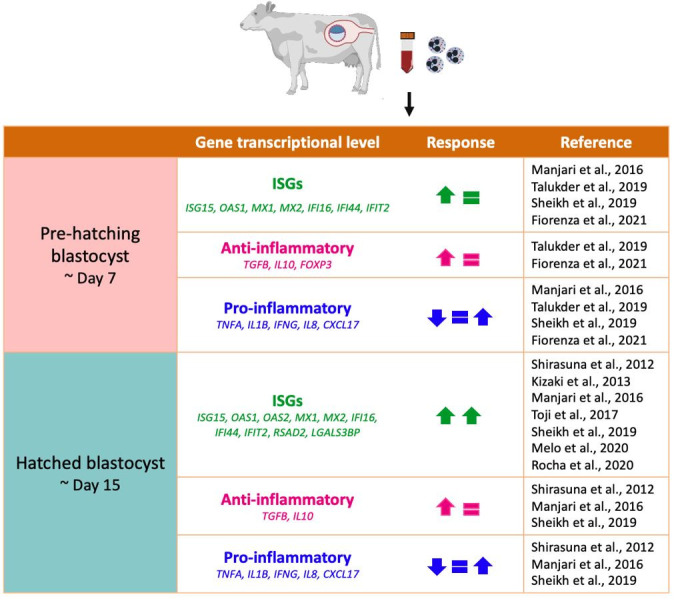
Summarized results from studies on cow’s PMN response. Upward and downward directed arrows indicate greater or lower expression, respectively. Equal signs indicate no changes in expression between control and treatment groups. Different colors denote different groups of markers.

The capacity of PMNs to orchestrate inflammatory and immune responses depends on their release of neutrophil-derived molecules, including cytokines, and their ability to interact with other innate and adaptive immune cells (Arck and Hecher, 2013). PMNs produce numerous anti- and pro-inflammatory cytokines (Mantovani et al., 2011) that are essential in pregnancy. In pregnant cows, PMNs showed an anti-inflammatory response with greater expression of TGFB, IL10, and forkhead box P3 (FOXP3) (Talukder et al., 2019). Pro-inflammatory cytokines data have shown biases in different studies; however, expression patterns seem to increase according to the development of pregnancy (Figure 1) (Fiorenza et al., 2021; Manjari et al., 2016; Sheikh et al., 2019; Shirasuna et al., 2012). Depending on their activation status, neutrophils can regulate other innate and adaptative immune cell activities and functions (Pillay et al., 2012) and IFNG production (Costantini and Cassatella, 2011). Also, PMNs can regulate Th1 and Th17 recruitment via the release of C-C motif chemokine ligand 2 (CCL2), chemokine (C-X-C motif) ligand 9 (CXCL9), and CXCL10 or CCL2 and CCL20, respectively (Pelletier et al., 2010), and induce T regulatory cells with proangiogenic phenotype demonstrated to aid pregnancy development (Nadkarni et al., 2016).

During early pregnancy, cows’ neutrophils had a higher concentration of IL10 and lowered IL8 on Days 14, 16, and 18. Pregnant cows had higher expression of ISGs (Kizaki et al., 2013; Rocha et al., 2020; Shirasuna et al., 2012), such as *ISG15*, 2’-5’-oligoadenylate synthetase 1 (*OAS1*), myxovirus resistant genes (*MXs*), interferon-gamma inducible protein (IFI) 16 (*IFI16*), and *IF144*. Non-pregnant cows had higher gene expression of cluster of differentiation (CD) *62L* (*CD62L*), *CD11b*, and *IL8* (Manjari et al., 2016). PMNs respond to IFNT around day 14 of pregnancy, earlier than other immune cells, and with much stronger gene expression (Melo et al., 2020; Shirasuna et al., 2012), implying that these cells are more sensitive to IFNT (Rocha et al., 2021; Toji et al., 2017). ISGs has antiviral, antiproliferative, and possibly immunosuppressive roles. ISGs can also be detected in PMNs earlier than traditional methods of pregnancy detection, such as ultrasound (Kizaki et al., 2013; Rocha et al., 2020; Toji et al., 2017; Yoshino et al., 2018); however, the sole use of gene expression still is not a feasible method to accurately detect pregnancy due to more significant false-negative and false-positive results (Dalmaso de Melo et al., 2020). Besides that, the IFNT signal regulates the expression of IL8 and ISG15 in PMNs. The effects of IL8 and ISG15 allow PMNs to infiltrate the CL to significantly increase P4 secretion during MRP (Shirasuna et al., 2015).

Polymorphonuclear cells contribute to conception, pregnancy establishment, and embryo protection (Giaglis et al., 2016). During pregnancy, oxidative burst and intracellular hydrogen peroxide production by PMNs were significantly decreased (Crouch et al., 1995). The crucial role in pregnancy was detected when PMNs depletion led to placental development impairment and reduced the number of viable offspring in mice (Higashisaka et al., 2018). PMNs may present two types of phenotypes in human pregnancy (Ssemaganda et al., 2014). These PMNs polarize from one type to another, depending on the stimuli, being classified as low-density neutrophils (LDN), representing anti-inflammatory response type, or high-density neutrophils (HDN), representing pro-inflammatory response type (Fridlender et al., 2009).

Low-density neutrophils promote tissue growth through cytokine secretion, increased angiogenesis, and extracellular matrix modulation (Granot and Jablonska, 2015). The LDN can be immature, derived from myeloid cells and mature cells (Sagiv et al., 2015). These PMNs have as characteristic the high expression of arginase-1 (ARG1), CCL2, CCL5, and vascular endothelial growth factor (VEGF), and the ability to inhibit T cell functions (Fridlender et al., 2009). Conversely, HDN limits cellular proliferation (Finisguerra et al., 2015). The HDN phenotype has a hyper-segmented nucleus, high expression of CCL3, CD54, and TNFA, and the ability to activate T cytotoxic cells (Fridlender et al., 2009). TGFB and granulocyte colony-stimulating factor (GCSF) modulate polarization to an anti-inflammatory phenotype (Casbon et al., 2015; Fridlender et al., 2009; Waight et al., 2011), while IFNB acts as a regulator of the pro-inflammatory phenotype (Jablonska et al., 2010; Wu et al., 2015). Stimulation of cows’ PMNs with IFNT *in vitro* generated an anti-inflammatory response by expressing *ISGs* and *TGFB*, CD16, and ARG1 (Fiorenza et al., 2021), well-known markers to maternal immune response suppression (Kropf et al., 2007).

**Figure 2:**
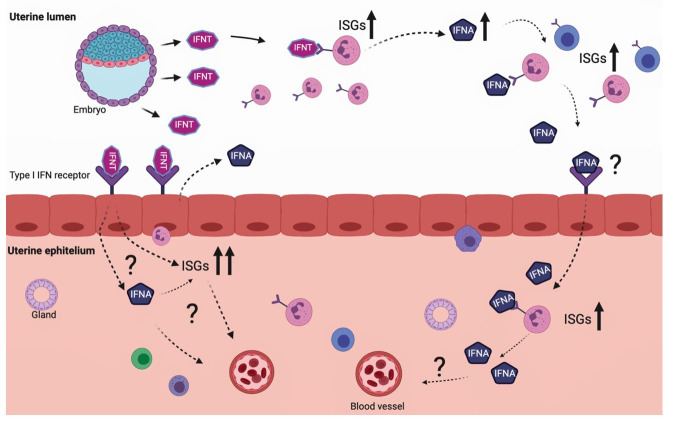
An additional model proposal for the role of immune cells in amplifying IFNT-derived signals. Embryos as early as Day 7 produce IFNT, inducing interferon-stimulated genes (ISGs) and interferons (IFNs), mainly IFNA in PMNs. Pre-hatching blastocyst secretes minutes concentration of IFNT that bindings to its receptors in the uterine epithelium stimulating ISGs, possibly to help maintain pregnancy. IFNT binds to its receptor on the surface of polymorphonuclear cells (PMNs) to stimulate the expression of ISGs and IFNA. After the initial stimulation, these local PMNs produce farther IFNA that have a similar response inducing the expression of ISGs in immune cells, possibly to induce embryo-maternal tolerance. The effects of PMN-release IFNA in uterine epithelial cells are still unclear. Solid upward-directed arrows indicate greater expression. Dotted arrows stated the release of molecules by the cells. The question mark represents the unclear functions of the molecules.

The ability to amplify and transfer IFNT signals to other immune cells is another essential function of PMNs during early pregnancy. The endocrine effects of IFNT appear around Day 15 of pregnancy. However, it’s unclear how the embryo and maternal immune system communicate earlier, proximately Day 7. PMNs express varied genes after IFNT priming, including type I IFNA and IFNB. In an *in vitro* experiment, PMNs were primed, washed to remove IFNT, and then re-incubated to comprehend if, even after removing IFNT from the system, PMNs could secrete different molecules to continue amplifying the IFNT signal to a different set of PMNs. As a result, PMNs had an IFN-like response with upregulation of ISGs and signal transducer and activator of transcription 1 (STAT1), implying that PMNs produce more IFNs, particularly IFNA, to amplify the IFNT signal around Day 7 (Figure 2) and create a tolerant environment during early pregnancy (Fiorenza et al., 2021).

Therefore, neutrophils significantly impact because of their ability to act as first responder cells even during pregnancy, transfer signals to other cells locally or systemically, and regulate their functions as a result.

## Perspectives

The maternal immune system plays a critical role in establishing, maintaining, and completing a successful pregnancy. However, the specific mechanisms to achieve these goals are not fully understood. Future studies may dictate the communication between mother and embryo and how the immune system can modulate the maternal immune response focusing on innate immune cells, such as PMNs. This communication might occur *via* soluble factors like cytokines, especially anti-inflammatory ones, such as TGFB, to generate a tolerant environment towards the embryo. However, lately, extracellular vesicles are gaining focus due to their ability to carry bioactive molecule as proteins, lipids, miRNAs, and mRNAs (Simpson et al., 2008; Subra et al., 2007; Valadi et al., 2007), which can modulate oocyte fertilization, embryonic development, and embryo-maternal communication, aiding pregnancy establishment (Bridi et al., 2020). Studies *in vitro* are necessary to fully understand the mechanism. *In vivo* studies are needed to confirm this hypothesis.
